# Sequencing and assembling bear genomes: the bare necessities

**DOI:** 10.1186/s12983-022-00475-8

**Published:** 2022-11-30

**Authors:** Courtney Willey, Ron Korstanje

**Affiliations:** The Jackson Laboratory, Bar Harbor, ME 04609 USA

**Keywords:** Ursidae, Bear, Genome, Gene expression, Sequence

## Abstract

Unique genetic adaptations are present in bears of every species across the world. From (nearly) shutting down important organs during hibernation to preventing harm from lifestyles that could easily cause metabolic diseases in humans, bears may hold the answer to various human ailments. However, only a few of these unique traits are currently being investigated at the molecular level, partly because of the lack of necessary tools. One of these tools is well-annotated genome assemblies from the different, extant bear species. These reference genomes are needed to allow us to identify differences in genetic variants, isoforms, gene expression, and genomic features such as transposons and identify those that are associated with biomedical-relevant traits. In this review we assess the current state of the genome assemblies of the eight different bear species, discuss current gaps, and the future benefits these reference genomes may have in informing human biomedical applications, while at the same time improving bear conservation efforts.

## The role of reference genomes for model and non-model organisms

A reference genome is a digital nucleic acid sequence database that is a representative example of a species and can be assembled from DNA sequences of various individuals. They can be used as a template to allow faster and cheaper assembly of the genomes of individuals from that same species or as a guide for gene expression studies. Reference genomes for model organisms have been important for the field of biomedicine by allowing for interspecies comparisons and linking specific genetic events to disease phenotypes [1–4]. Reference genomes also serve a role in understanding the biological significance of model organisms such as the mouse (*Mus musculus*) [5], the fruit fly (*Drosophila melanogaster*) [6], and the zebrafish (*Danio rerio*) [1]. Although, common model organisms such as these are standard in the biomedical field, it does not mean that other organisms do not have biomedical relevance.

The growing number of reference genomes from non-model organisms has revolutionized a myriad of scientific fields with applications in biomedicine and conservation. Various organisms have unique adaptations that may inform the development of novel treatments for disease in humans [7, 8]. For example, the reference genomes of African and Asian elephants (*Loxodonta africana* and *Elephas maximus indicus*, respectively) have revealed differential inflammatory responses for disease defense and provided important evolutionary insights that may be translated into human patients with infections or cancer [9]. The Antarctic Weddell seal’s (*Leptonychotes weddellii*) reference genome revealed evolutionarily favored genes that affect the cardiovascular system, regulation of hypoxia signaling and lipid synthesis, and transport mechanisms [10]. These findings hold biomedical potential for humans who would likely develop cardiovascular disease, from a similar high-lipid diet, lifestyle, and risk factors.

## Applications in conservation

**Fig. 1:**
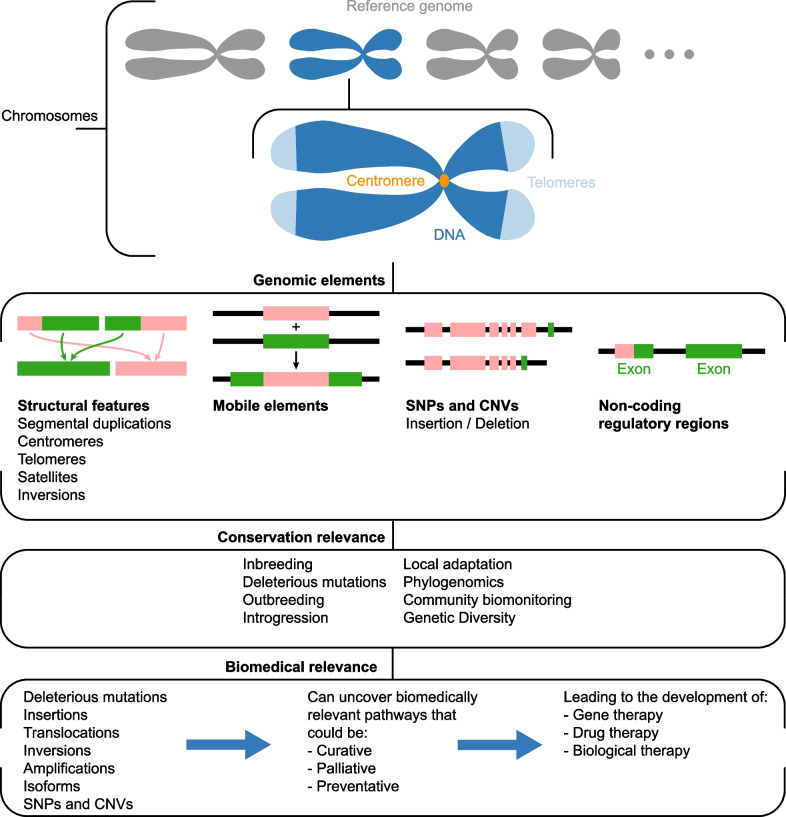
Structural features of a reference genome. Reference genomes can help characterize these genomic structures, especially if they are not fragmented. Variations in these structures can then help inform both conservation and biomedicine. Based on a figure by Formenti et al. [11]

Mammalian genomes are sequenced for different reasons, yet efforts are typically focused upon conservation as reference genomes can be used to uncover valuable information about genomic diversity (inbreeding, deleterious mutations, outbreeding, introgression, local adaptation) that aid in conservation management strategies (phylogenomics and community biomonitoring) (Fig. 1) [11, 12]. For example, modern day reference genomes for the Grauer’s gorilla (*Gorilla beringei graueri*) and the small mountain gorilla (*Gorilla beringei beringei*) compared to whole genomes from historical museum specimens revealed different genetic responses to population decline [13]. Another example, the Tasmanian devil (*Sarcophilus harrisii*), is threatened by a deadly transmissible cancer called devil facial tumor disease (DFTD) and its genome has revealed adaptive genetic variation at loci associated with DFTD as well as rapid evolution in response to DFTD [14]. As for bears, the Andean bear (*Tremarctos ornatus*), also known as the spectacled bear, Asiatic black bear (*Ursus thibetanus*), giant panda (*Ailuropoda melanoleuca*), polar bear (*Ursus maritimus*), sloth bear (*Melursus ursinus*), and sun bear (*Helarctos malanus*) are currently listed by the International Union for Conservation of Nature (IUCN) as vulnerable [15].

## Why sequence the bear genome?

**Fig. 2:**
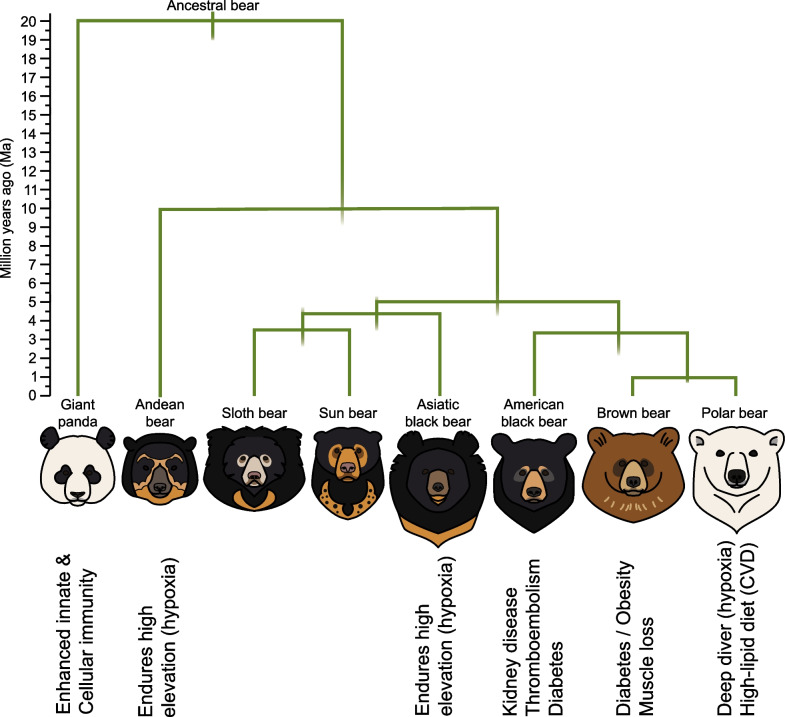
Biomedically Relevant Bear Traits and Phylogenetic Tree. Bears traits shape biomedical applications. Each bear mentioned here has a unique trait, that could benefit the field of biomedicine. The Ursidae family phylogenetic tree has been shaped by the help of reference genomes. The initial divergence within bears begins with the giant panda (*A. melanoleuca)* followed by the Andean bear (*T. ornatus*). The Asiatic black bear (*M. thibetanus*) is sister to the sun bear (*M. malayanus*) and sloth bear (*M. ursinus*), while the American black bear (*U. Americanus*) is sister to the brown bear (*U. arctos*) and polar bear (*U. maritimus*). Based on a figure by Kumar et al. [37]

Like many other species, bears have unique traits that are biomedically relevant. These traits were developed in response to the environment, diet, and hibernation status. Extant bears exhibit enormous variability regarding their environment, diet, and hibernation status, offering many traits to explore (Table 1 and Fig. 2). To make significant inroads into understanding these unique traits and their underlying molecular mechanisms, there is a need for high-quality, well-annotated reference genomes of the different bear species.

**Table 1:** Important features and traits for each species

Bear	Environment	Diet	Hibernator	Unique Traits	Human relevance	References
Giant panda	Broadleaf & coniferous, temperate forests	Herbivorous	No	Enhanced innate & cellular immunity	Autoimmune diseases	[27]
Brown bear	Variety of habitats (from desserts, to mountains, to tundra)	Omnivorous	Yes	Protects vital metabolic functions during hibernation	Diabetes, obesity, muscle loss	[23–25, 29]
Asiatic black bear	High-elevation forested hills & mountains	Omnivorous	Yes	Endures hypoxic conditions & protects vital metabolic functions During Hibernation	Asthma, lung disease, anemia	[17, 30]
Andean bear	High-elevation forested hills & mountains	Omnivorous	No	Endures hypoxic conditions	Asthma, lung disease, anemia	[18]
Polar bear	Arctic	Carnivorous	Females only	Endures hypoxic conditions & a high-fat diet	Cardiovascular disease, asthma, lung disease, anemia	[19, 20]
American black bear	Coniferous & deciduous forests & open alpine habitat	Omnivorous	Yes	Protects vital metabolic functions during hibernation	Kidney disease, venous thromboembolism, diabetes	[21, 22]
Sun bear	Tropical rain forests	Omnivorous	No	N/A	N/A	N/A
Sloth bear	Wet & dry tropical forests	Omnivorous	No	N/A	N/A	N/A

Different environments create unique challenges that bears have adapted to for survival. For example, the Asiatic black bear (*Ursus thibetanus*) and the Andean bear (*Tremarctos ornatus*), evolved to endure hypoxic conditions at high elevation [16–18], while polar bears (*Ursus maritimus*) endure hypoxic conditions when diving under-water for prey [19]. Understanding the genomic mechanisms behind these traits could inform the treatment of hypoxic conditions such as asthma, lung disease, or anemia.

Bears have also developed unique dietary adaptations, that may provide insights into our own diets and how it affects our health. Most bear species are omnivorous, except for the carnivorous polar bear and the herbivorous giant panda (*Ailuropoda melanoleuca*). The polar bear can protect its heart and vascular system from high low-density lipoprotein (LDL) levels, in response to their hyper-lipid diet of marine mammals [19, 20]. This suggests the polar bear’s diet may reveal useful information for human health, due to their unique ability to protect their heart and vascular system from the consequences of a high-fat diet.

The environment also determines whether or not some bear species hibernate, and determines how their metabolic system works, making hibernating bears (Asiatic black bear, American black bear (*Ursus americanus*), and brown bear (*Ursus arctos*)) of interest for studying metabolic diseases and “burden of lifestyle” diseases in humans (coronary heart disease, cancer, congestive heart disease, chronic kidney disease, obesity, type-2 diabetes, and non-alcoholic fatty liver disease) [7] as they first increase their metabolism to prepare for hibernation and subsequently lower it during hibernation [21–25]. Non-hibernating bears (sun bear (*Helarctos malayanus*), sloth bear (*Melursus ursinus*), giant panda, and polar bear) also offer unique opportunities by providing genetic information related to a non-sedentary lifestyle.

## Reference genomes have revealed biomedical relevance

### Giant panda

The giant panda reference genome has provided insight to their unique innate and cellular immunity. RNAseq was used to obtain transcriptomic information for the giant panda’s heart, liver, lung, and kidneys and was compared to previous transcriptome information for the blood and spleen [26]. Expression profiles associated with immunity revealed that breeding male giant pandas displayed enhanced innate immunity and cellular immunity with lower humoral immunity compared to non-breeding males, and identified 45 immune-related genes with altered expression (mostly up-regulated) [27]. In addition, the giant panda genome seems to have maintained the genetic requirements for being purely carnivorous, but a mutation in *T1R1*, that encodes a protein involved in sensing savory flavors might be (in part) why the panda prefers bamboo. It has also been found that bamboo is very rich in protein and low in carbohydrates meaning the nutritional content of bamboo is similar to meat [28]. However, the panda lacks genes needed for bamboo digestion, suggesting a large role of its microbiome [28].

### Polar bear

The polar bear is a marine Arctic dweller capable of swimming underwater and staying submerged for several minutes, a highly demanding lifestyle that holds biomedical potential for humans [19]. Such a high-energy lifestyle relies on selection in genes related to the production of nitric oxide (*NOS3*, *CPS1*, *TLR4*, *CAV3*, and *ENG*), a vasodilator, playing the role of an energetic gatekeeper. [19]. Though genomic analyses have not identified any enriched pathways linked specifically to hypoxia tolerance or hypoxia response signaling, there were changes in gene expression related to blood pressure [19]. In order to make sure the heart and brain are protected from apnea-induced hypoxemia during deep dives, a cardiovascular adjustment occurs in which peripheral arterial beds are more constricted [7]. This supporting evidence means polar bears have excellent cardiovascular control necessary for diving response, despite having a hyper-lipid marine mammal diet that would put humans at high risk for cardiovascular disease [19].

### Brown bear

The genomes of hibernating bears such as the brown bear provide key information related to metabolic function. During hibernation (about 6 months out of the year), both brown bears and black bears lower their blood flow and heart rate to as low as 10 beats per minute to protect their bodies from physical inactivity [24, 29]. Humans cannot do this and therefore suffer from the consequences of physical inactivity, often an increased risk of developing a “burden-of-lifestyle disease such as venous thromboembolism (VTE) [29]. Comparing physical inactivity and platelet function in humans and brown bears has revealed that three weeks of bed rest had no effect on human platelet aggregation levels, but in hibernating brown bears, platelet aggregation was halved compared to summer [29]. This is believed to be a protective measure to avoid the formation of thrombi during periods of physical inactivity when circulation is decreased [29]. Interestingly, in late summer and fall, brown bears also gain up to 30% in body mass compared to in the spring and preserve insulin sensitivity (a feat humans cannot achieve) [24].

### American black bear

Like the brown bear, the American black bear has evolved features in the kidney that allow them to endure low renal function during hibernation and recover it soon after hibernation [21]. In 2019, a set of 169 differentially expressed genes that may be involved in the American black bear’s unique hibernation response were identified [21]. Specifically, the upregulation of three cytokine suppression genes were found after hibernation (*SOCS2*, *CISH*, and *SERPINC1*), while there was a lack of increased cytokine expression (*IL6, CCL2, CCL6*) and damage markers (*LCN2* and *HAVCR1*) normally found in low functioning or recovering kidneys in other species [21].

### Asiatic black bear

Humans would typically suffer severe pulmonary hypertension when exposed to hypoxic conditions in high elevations yet, the Asiatic black bear is able to endure these conditions without any serious repercussions [17]. The Asiatic black bear lives predominantly in forested hills and mountains at elevations over 9,900ft in the summer and descend lower in the winter to prepare for the cold [30].

### Andean bear (spectacled bear)

Like the Asiatic black bear, the Andean bear is adapted to high elevations. However, data is lacking on the Andean bear due to poaching and other threats which have limited studies concerning genetics and led to a focus on conservation methods [18].

## Current gaps and future benefits

Currently, there is a lack of high-quality reference genomes for bears and some species are completely missing a reference genome (Table 2), creating gaps that are affecting the quality of research that can be accomplished. Some of the current limitations of Ursidae biomedical applications include the possibility of false positives that can occur during genome-wide analysis, as well as the lack of experimental validation, and how specific mechanisms can be turned into biomedical practices. These limitations provide roadblocks to achieving biomedical applications, however, these limitations may be remedied if more high-quality reference genomes are obtained. As more high-quality reference genomes are obtained and the number of reference genomes for a species increases, the possibility of false positives is reduced, and the lack of experimental validation is solved through comparison of genomes.

**Table 2:** Summary of genome assemblies

Species	Sequencing technology	Size (Gbp)	N50 (bp)	# Scaffolds	Chr (2n)	NCBI Acc. #	References
Giant panda	Illumina; 10X Genomics; Flow-sorted chromosome sequencing	2.4	129,200,000	73,513	42	PRJNA588422	[36]
Brown bear	PacBio; Illumina	2.5	70,100,000	901	74	PRJNA807323	[23]
Asiatic black bear	Illumina NovaSeq	2.4	26,800,000	24,967	74	PRJNA573607	[17]
Andean bear	Illumina; Oxford Nanopore	2.2	21,100,000	12,402	52	PRJNA472085	[18]
Polar bear	Ilumina HiSeq; Oxford Nanopore	2.3	72,000,000	3899	74	PRJNA669153	
American black bear	PacBio	2.4	13,900,000	2212	74	PRJNA319796	[21]
Sun bear	N/A	N/A	N/A	N/A	74	N/A	
Sloth bear	N/A	N/A	N/A	N/A	74	N/A	

A good measure of the quality of a genome is scaffold-level contiguity [18]. Genome assemblies using a combination of techniques such as short read, long read, and proximity ligation data produce better quality assemblies (giant panda, NCBI Accession #PRJNA588422) than ones that purely rely upon one method such as short read (American black bear, NCBI Accession #PRJNA31976) [18]. Also, fragmented, or incomplete genomes cannot be used to accurately characterize structural features of a genome [11]. Structural features of a genome include the building blocks of chromosomes (centromeres, telomeres, satellites, and structural variations such as rearrangements and segmented duplications) (Fig. 1). Generating complete, highly contiguous, correctly annotated genomes for all Ursidae using long-read sequences will allow us to identify differences in genetic variants, isoforms, gene expression, and genomic features like transposons (see Glossary). These differences may lead to altered gene function and subsequent protein production, allowing for the identification of novel genes for biomedical research and conservation. Therefore, focusing on obtaining high-quality reference genomes, will improve the tools needed to identify genomic changes between species, and better inform biomedicine and conservation efforts. However, the possibility of false positives and the lack of experimental validation remains unless a larger number of high-quality reference genomes are produced for each species to allow for comparative and forward genomics.

Obtaining multiple high-quality genomes of Ursidae members, will improve current gaps in reference genome quality and annotations, resulting in clearer evolutionary history and allowing for higher quality gene expression studies, comparative genomics, and forward genomics to better inform conservation and human biomedicine, reducing the instance of false-positives and allowing for the discovery of experimental validation methods [2–4, 31]. Comparative genomics is a field of research in which complete genome sequences are compared between different species, while forward genomics is an approach to determine the genetic underpinnings responsible for a certain phenotype. Comparative and forward genomics highlight the need for high-quality reference genomes for all mammals to achieve a deeper understanding of which genetic variants lead to certain phenotypes, especially disease phenotypes, and how we can predict these changes [2–4, 31]. Both, comparative and forward genomics also utilize multiple reference genomes to track when certain variants arose within a species, which can help designate evolutionary significance that dictates conservation priorities. Being able to pinpoint specific variants and genes related to disease phenotypes can help identify therapeutic targets for drug design in both humans and mammals (see Fig. 2) [2, 4]. As more high-quality genomes are produced and can be compared, false positives will be more readily identified allowing for some biological mechanisms to have a more obvious role.

Genome assembly and annotation can also be improved with the help of software like Progressive Cactus, an extension for Cactus, that allows for multiple large vertebrae genomes to be aligned while maintaining high quality [32]. Aligning sequences helps to identify similar regions of DNA for easy comparison of genome sequences between multiple large vertebrates, making Progressive Cactus a great addition to the field of comparative genomics. Aligning and analyzing extinct and extant genomes may even help explain discordances in evolutionary history and answer how evolution impacted the flow of genetic information on extant Ursidae members (see Fig. 2) [33, 34]. This also proves useful for identifying the genetic history of evolutionarily conserved traits that could hold biomedical importance.

A next step in resolving genomes, after obtaining complete high-quality reference genomes, is assigning contigs and scaffolds to individual chromosomes (see Glossary). Most bear species have 36 autosomes and two sex chromosomes, except for the giant panda (20 autosomes and two sex chromosomes) and the Andean bear (25 autosomes and two sex chromosomes). Chromosomal assignment will be useful for population genetic studies, as it allows selection of genetic markers that we know will be unlinked, and it can provide more insight into the evolution of bear species by studying chromosomal rearrangements. Assigning contigs and scaffolds to the sex chromosomes can be achieved through male/female sequence comparison and sequence comparison with other mammalian species. Once high-quality genome assemblies have been obtained for all eight Ursidae species and have been assigned to different chromosomes, the next step would be to move onto obtaining the same information for all Ursidae subspecies. This would then provide a deeper genomic insight to the relationships between bears and how their unique traits came to be, further reducing gaps and limitations.

Currently available reference genomes for Ursidae show promise in uncovering future biomedical application in humans (Table 1 and Fig. 2). Understanding the biological mechanism that is responsible for enhanced innate immunity and cellular immunity in giant pandas may provide new methods of treatment for our own innate and cellular immunity [27]. Further studies related to the biology behind the polar bear’s cardiovascular system and high-energy lifestyle could uncover biomedically relevant information useful for treating humans with cardiovascular diseases [19, 20]. Because brown bears and American black bears are evolutionarily adapted to periodic obesity and long periods of physical inactivity, their genomes may hold keys to prevention and treatment strategies for metabolic and “burden-of-lifestyle” diseases in humans such as CKD and VTE [21–25, 29]. In regards to the Asiatic black bear and Andean bear, a genomic study in humans adapted to the high elevation in Tibet (11,995ft), revealed three novel genes *EPAS1, EGLN1,* and *PPARA* that are strongly associated with lower hemoglobin concentration, increased lactate production, and decreased fatty acid oxidation [35]. It would be interesting to see if these same genes play a role in the Asiatic black bear and Andean bear or whether other genes allow for their adaptation to high elevation. This can help inform biomedical treatment of hypoxia related disease in humans (asthma, lung disease, and anemia) [17, 18, 30]. Uncovering these biomedical mechanisms is currently underway, and with future improvement to reference genome technology and the number of genomes obtained there is promise for biomedical applications in humans.

## Conclusions

Genomic resources such as reference genomes are important for uncovering the unique traits bears have due to variable environments, hibernation, and diets that may have important biomedical applications for humans. So far, reference genomes for the giant panda, polar bear, brown bear, American black bear, Asiatic black bear, and the Andean bear (Table 2) have provided evidence that their unique traits may be useful for the prevention and treatment of many diseases, mostly those of the metabolic system. Based on the potential of what we have seen so far, generating high-quality reference genomes for all bears is beneficial for the conservation of bears vulnerable for endangerment, advancement of human biomedicine, and overcoming the current limitations hindering biomedical applications in humans.

## Data Availability

Not applicable.

## Abbreviations

LDL: Low-density lipoprotein
NCBI: National Center for Biotechnology Information
Gbp: Billion base pairs
bp: Base pairs
VTE: Venous thromboembolism
CKD: Chronic kidney disease
DFTD: Devil facial tumor disease

## Glossary

N50: The N50 value is calculated by first ordering every contig/scaffold by length from longest to shortest. Next, starting from the longest contig/scaffold, the lengths of each contig are summed, until this running sum equals one-half of the total length of all contigs/scaffolds in the assembly. This is used to measure the completeness of a genome
Scaffold: A series of non-contiguous DNA sequences linked together
Transposon: Also known as a transposable element, is a DNA sequence that can change position within a genome, potentially creating or reversing mutations that alter genetic identity and genome size
